# Effect of Artificial Saliva Modification on Pitting Corrosion and Mechanical Properties of the Remanium^®^-Type Orthodontic Archwire

**DOI:** 10.3390/ma16206791

**Published:** 2023-10-20

**Authors:** Bożena Łosiewicz, Patrycja Osak, Karolina Górka-Kulikowska, Tomasz Goryczka, Michał Dworak, Joanna Maszybrocka, Krzysztof Aniołek

**Affiliations:** 1 Institute of Materials Engineering, Faculty of Science and Technology, University of Silesia in Katowice, 41-500 Chorzow, Poland; patrycja.osak@us.edu.pl (P.O.); tomasz.goryczka@us.edu.pl (T.G.); michal.dworak@us.edu.pl (M.D.); joanna.maszybrocka@us.edu.pl (J.M.); krzysztof.aniolek@us.edu.pl (K.A.); 2 Department of Biomaterials and Experimental Dentistry, Poznan University of Medical Sciences, 60-812 Poznan, Poland; karolinagorka.profil@gmail.com

**Keywords:** electrochemical impedance spectroscopy, Fe–Cr–Ni steel, pitting corrosion, Remanium orthodontic archwire, saliva

## Abstract

The pitting corrosion of orthodontic apparatus elements in the oral environment is an interest of both clinicians and scientists dealing with the assessment of the biocompatibility of medical materials. This work presents a study on the effect of ready-to-use Listerine^®^ and Meridol^®^ mouthwashes and sodium fluoride on the resistance of the commercial Remanium^®^-type orthodontic archwire to pitting corrosion in artificial saliva at 37 °C. XRD, SEM, EDS, mechanical properties, and microhardness measurements were used to characterize the archwire. The in vitro corrosion resistance of the archwire was examined using the open-circuit potential method, electrochemical impedance spectroscopy, and anodic polarization curves. The physicochemical characteristics confirmed the presence of a bi-phase alloy with a mixed austenite/ferrite structure containing Fe 74.4(7) at.%, Cr 18.4(4) at.%, and Ni 7.2(4) at.%. The Fe–Cr–Ni alloy was characterized by high tensile strength and Vickers microhardness. EIS revealed the capacitive behavior with high corrosion resistance. It was found that the kinetics of pitting corrosion in the artificial saliva decreased in the presence of NaF and mouthwashes. The potentiodynamic characteristics confirmed the decrease in susceptibility to pitting corrosion after the modification of artificial saliva. The pitting corrosion mechanism of the self-passive oxide layer on the surface of the Fe–Cr–Ni electrode in the biological environment containing chloride ions was discussed in detail. Mechanical properties after corrosion tests were weakened.

## 1. Introduction

In dental prosthetics, metallic biomaterials are widely used, and include high-noble alloys, noble alloys, and non-noble alloys [1,2,3,4,5,6,7,8,9,10,11]. High-noble alloys contain 60% of precious elements such as Au, Pt, Pd, Ir, and Ru and a minimum of 40% Au [1]. Noble alloys contain 25% by weight of precious elements, not necessarily gold. Base alloys mainly contain base metals such as nickel, chromium, cobalt, niobium, and titanium. The noble metal content does not exceed 25% by weight of the base alloy. However, prosthetic constructions made of dental biomaterials are subject to electrochemical phenomena in the oral cavity, which may adversely affect the human body. Corrosion of dental biomaterials can lead to weakening of the prosthetic restoration and, consequently, to its damage [1,2,3,4,5,6,7,8,11].

In the case of orthodontic wires working in the oral cavity environment, corrosion is caused by the strong corrosive properties of saliva associated with the presence of chloride ions, proteins, enzymes, and bacteria on the dental plaque [1,3]. Bacteria make metals and their passivation alloys unable to prevent biological corrosion. Additional factors of corrosion are changes in pH caused by consumed foods, diseases such as gastric hyperacidity or gastroesophageal reflux disease, changes in the temperature of drinks, and errors made during the production of alloys and their thermal and mechanical treatment.

Metal ions are released during the corrosion process and their content in the saliva increases over time [3,12,13,14,15]. Metal ions can also be detected in the gums and tissues adjacent to the inserted dental alloy, they also accumulate in the stomach, liver, and kidneys. Each metal has a critical concentration beyond which toxic and allergic reactions occur. The degree of harmful effect of dental alloys depends mainly on the amount of released corrosion products.

Very durable and resilient Fe–Cr–Ni Remanium^®^ steel belongs to non-noble alloys, which is widely used in dentistry, especially in orthodontics, jaw orthopedics, and prosthetics due to its acid-resistant properties [14,15,16]. The content of chromium and nickel in alloys for dental applications is regulated by the ISO 6871-2:2000 standard [17]. Both chromium and nickel undergo self-passivation, thanks to which Fe–Cr–Ni steel shows good resistance to quite aggressive corrosive environments of the oral cavity.

The gradient formed during corrosion at the metal-intercellular fluid interface weakens the activity of neutrophils, macrophages, and lymphocytes, which weakens the activity of the positive bacterial flora [14,15,16,17,18,19,20]. Small amounts of bioelements in the body are needed; however, too high a level resulting especially from electrochemical corrosion can lead to many diseases, which include electrometallosis and ion poisoning. Excess iron formed as a result of corrosion accumulates in the tissues surrounding the alloy and in the cells of the spleen. Iron ions destroy lysosomes, thus hindering the diffusion of enzymes through cell membranes. The excess of iron ions leads to the formation of free radicals and, consequently, to DNA and RNA damage, mutagenic changes, and atherosclerosis of the vessels [18]. Chromium ions as a product of the corrosion of dental alloys, also cause changes in the tissue in direct contact with the alloy. Their concentration may be even 30- to 100-fold higher than that of normal tissues. The accumulation of chromium ions causes damage to parenchymal organs, such as kidneys and liver. Excess chromium ions are also deposited in the spleen. Chronic exposure to chromium also causes allergic reactions, mainly in men, and carcinogenic reactions [19]. Nickel is a non-toxic element, but nickel tetracarbonyl is highly toxic as it damages the bronchial mucosa. Nickel ions are carcinogenic and cause allergic reactions mainly in women. Metal ions are accumulated in the spleen and kidneys. Elevated nickel concentration is also observed in the tissues near the alloy [20]. The listed bioelements as corrosion products in the form of ions move into the tissues with body fluids, damaging the cell membrane, which changes the metabolic activity of the cells. Metal ions can affect the intercellular space on cells or penetrate into them also by phagocytosis. As a result of corrosion in soft tissues, phagocytic staining can be observed. High corrosion resistance is a prelude to biocompatibility [21].

This work addresses issues that are extremely important from the application point of view, which concern a very wide group of patients around the world who use the Fe–Cr–Ni orthodontic archwire of the Remanium^®^-type exposed to aggressive factors during the treatment process. These include the acidic pH of saliva caused by inflammation or the intake of fluids and food, the presence of commercial water- and alcohol-based mouthwashes, and the presence of sodium fluoride, which is the main ingredient of toothpastes. The research undertaken was carried out in cooperation with practicing dentists and prosthodontists. This work provides new insight into the relationship between corrosive factors present in saliva and local pitting corrosion of commercial orthodontic wires of the Remanium^®^ type. In vitro corrosion resistance testing was performed using electrochemical impedance spectroscopy (EIS) to characterize the mechanism and kinetics of electrochemical corrosion. The assessment of corrosion damage after the potentiodynamic measurements was carried out using scanning electron microscopy (SEM) and energy dispersion spectroscopy (EDS). Mechanical properties and microhardness of the tested archwire were also the subject of this study.

## 2. Materials and Methods

### 2.1. Preparation of Samples for Testing

**Figure 1:**
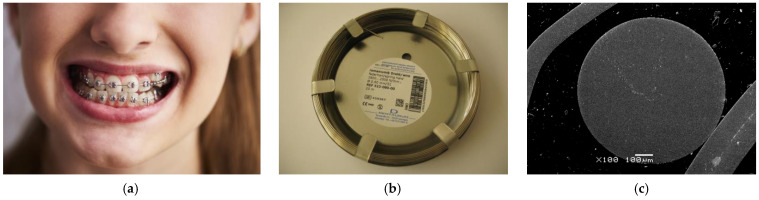
Dentaurum Remanium^®^ archwire: (**a**) in the orthodontic apparatus as arches [22]; (**b**) in trading condition; (**c**) SEM image of the sample after inclusion in graphite.

The research material was a commercial orthodontic archwire of the Remanium^®^ type (Dentaurum, Ispringen, Germany), made of Fe–Cr–Ni steel with a diameter of 0.80 mm and a length of 20 m, which is used for ready-made wire elements for assembly in removable orthodontic appliances (Figure 1). The round, spring-hard Remanium^®^ archwire was cut into 50 mm long samples. The Remanium archwire samples were also collected in graphite using an ATM Opal 400 hot mounting press (Spectrographic Ltd., Guiseley, Leeds, UK) using a pressure of 3.5 bars at 180 °C for 10 min to determine the microhardness of the tested alloy. Then, the collected samples were ground with the metallographic grinding and polishing machine Metkon Forcipol 102 (Metkon Instruments Inc., Bursa, Turkey) on abrasive papers with a gradation from P320 to P2500 (Buehler Ltd., Lake Bluff, IL, USA) and polished on felt with a colloidal SiO_2_ suspension (0.04 μm grain size, Struers, Cleveland, OH, USA), until the mirror surface was obtained. All samples were degreased in acetone (Avantor Performance Materials Poland S.A., Gliwice, Poland) for 20 min using an ultrasonic cleaner USC-TH (VWR International, Radnor, PA, USA). In the final stage, sonication in ultrapure water (Milli-Q Advantage A10 Water Purification System, Millipore SAS, Molsheim, France) for 20 min was applied.

The Dentaurum Remanium^®^ archwire belonging to medical devices Class IIa was easy to bend and could be welded [23]. Its composition was in at.%: Cr 16.0–19.0; Ni 6.0–9.5; Si ≤ 2.0; Mn ≤ 2.0; Mo ≤ 0.8; C 0.05–0.15; N ≤ 0.11; P ≤ 0.045; S ≤ 0.015; rest Fe. The Young’s modulus was equal to 134.24 Gpa.

### 2.2. Phase Composition Identification of the Fe–Cr–Ni Steel

X-ray diffractograms for the Fe–Cr–Ni steel were recorded using a Philips X’Pert PW3050 X-ray diffractometer (Malvern Panalytical, Worcestershire, UK) supplied with a current of 30 mA at a voltage of 40 kV, and a bent graphite monochromator. The Bragg–Brentano geometry and CuKα radiation (λCuKα—1.54178 Ǻ) was used. The registration was made using the step-scanning method with a step of 0.04° and a counting time of 4 s in the 2θ angular range from 20 to 140°. The slit on the incident beam was 1° and on the diffracted beam 1°. The Soller’s slits of 0.03 mm were used. The obtained diffraction pattern was the basis for phase identification using the ICDD PDF4 database (Release 2018) [24].

### 2.3. Microstructure and Chemical Composition of the Fe–Cr–Ni Steel

Microstructure studies of Fe–Cr–Ni steel were performed using a JEOL JSM-6480 scanning electron microscope (SEM, Peabody, MA, USA) with a resolution of 3 nm and an accelerating voltage of 20 kV. The analysis of the chemical composition of the samples before and after corrosion tests was carried out using the EDS method. The study of the surface distribution of alloying elements such as Fe, Cr, and Ni was also performed.

### 2.4. The Corrosion Resistance of the Fe–Cr–Ni Steel

The in vitro corrosion resistance of the Fe–Cr–Ni steel to pitting corrosion was tested in artificial saliva prepared according to the AFNOR/NF standard S90-701 [25] at pH 7.4(1) and pH 5.5(1) (Table 1). Artificial saliva solutions were enriched with 0.1 M NaF and 15 mL of commercial antiseptic mouthwashes such as Listerine Total Care Teeth Protection^®^ (McNeil Consumer Healthcare McNeil-PPC, Inc., Fort Washington, PA, USA) based on alcohol (21.6% *v*/*v*) and Meridol^®^ (Colgate-Palmolive Company, New York, NY, USA) alcohol free. For comparative purposes, corrosion resistance tests were also carried out in a 3.5% NaCl solution with pH 7.4(1) in accordance with the ISO 10271:2021 standard [26]. A 4% NaOH solution and a 1% C_3_H_6_O_3_ solution were used to adjust the pH of the solutions. Ultrapure water with a resistivity of 18.2 MΩ cm and reagents pure for chemical analysis (Avantor Performance Materials Poland S.A., Gliwice, Poland) were used to prepare all solutions.

In electrochemical measurements, the working electrode (WE) was a sample of the Remanium^®^ orthodontic archwire, and the counter electrode (CE) was a platinum foil. Electrochemical tests were conducted against a reference electrode (RE) in the form of a saturated calomel electrode (SCE) type R-20 (Hydromet, Gliwice, Poland), containing a half-cell with the scheme (Pt)Hg/Hg_2_Cl_2_ with the addition of a saturated solution of potassium chloride. RE was introduced into the electrolyte through a Luggin capillary.

In situ corrosion resistance tests were carried out in thermostatic conditions at 37(2) °C. Each solution was deaerated in 99.9999% pure argon for 30 min before measurement. The Autolab/PGSTAT12 (Metrohm Autolab B.V., Utrecht, The Netherlands) was used for the corrosion tests. The measurement using the open-circuit potential and the polarization curve method was performed by the ISO 10271:2021 standard [26]. The open-circuit potential (E_OC_) was recorded for a time (t) of 2 h.

EIS measurements were carried out at the E_OC_ in the frequency (f) range from 20 kHz to 10 mHz using the sinusoidal signal amplitude of 10 mV. EIS data were analyzed based on electrical equivalent circuits using the EQUIVCRT program and the complex non-linear least squares (CNLS) method. Equivalent circuits were defined according to the circuit description code proposed by Boukamp [27].

The anodic polarization curves were recorded from a potential 150 mV lower than the stabilized Eoc with a polarization rate of 1 mVs^–1^ to the value of 1 V.

### 2.5. Mechanical Properties of the Fe–Cr–Ni Steel

The mechanical properties of the Fe–Cr–Ni steel were investigated before and after corrosion investigations in the tensile test at ambient temperature using an Instron 1195 universal testing machine (Instron, Norwood, MA, USA) equipped with a video extensometer. The samples were 25 mm long and had a diameter of 0.80 mm. The beam displaced was at a rate of 1 mm min^–1^.

The micromechanical properties of the Fe–Cr–Ni steel were studied in the microhardness test using a Wilson^®^–WolpertTM Microindentation Tester 401MVD (Wilson Instruments, LLC, Carthage, TX, USA). The Vickers method with a hardness scale of HV = 0.1 and a Vickers indenter in the form of a square-based pyramidal-shaped diamond indenter with face angles of 136° was used according to the ISO 6507-1:2018 standard [28]. The maximum indentation load was 0.3 N for 10 to 15 s. For checking and calibrating the microhardness tester, indenter, and diagonal length of the measuring system, a direct method was used, which is described in the ISO 6507-2:2018 standard [29].

## 3. Results and Discussion

### 3.1. Physicochemical Characteristics of the Fe–Cr–Ni Steel

**Figure 2:**
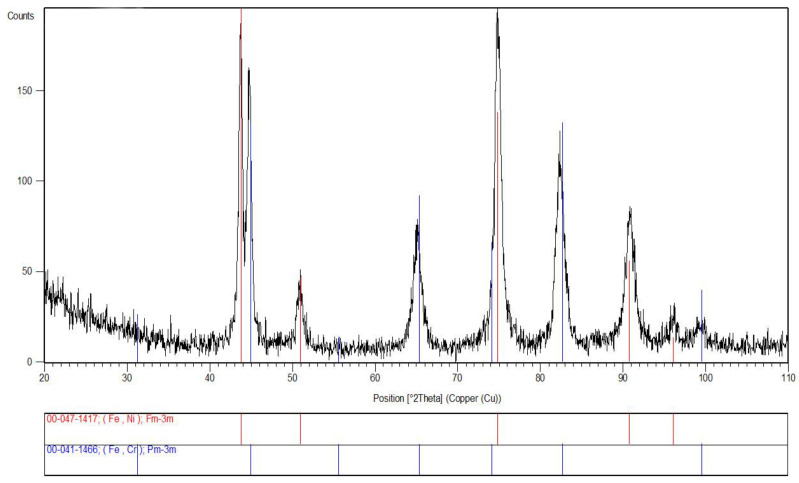
XRD diffractogram of the commercial Fe–Cr–Ni steel.

To confirm the phase composition of the Fe–Cr–Ni steel used for the production of the Remanium^®^-type orthodontic archwire, an X-ray structural analysis was conducted. Figure 2 presents an exemplary XRD diffractogram in the Bragg–Brentano geometry obtained for the commercial sample of the Fe–Cr–Ni steel. The phase identification carried out revealed that the registered diffraction lines are characteristic of ferrite Fe(Cr) (ICDD 00-041-1466) and austenite Fe(Ni) (ICDD 00-047-1417). Based on the X-ray analysis, it was found that the tested Fe–Cr–Ni steel is a bi-phase alloy with a mixed austenitic/ferritic (γ/δ) structure.

The obtained results suggest that the examined Fe–Cr–Ni still will maintain high resistance to pitting, intercrystalline, atmospheric, and stress corrosion, maintaining the high level of strength properties of ferritic stainless steel and a relatively low heat expansion coefficient compared to austenitic stainless steel, together with less tendency to grain growth [30].

**Figure 3:**
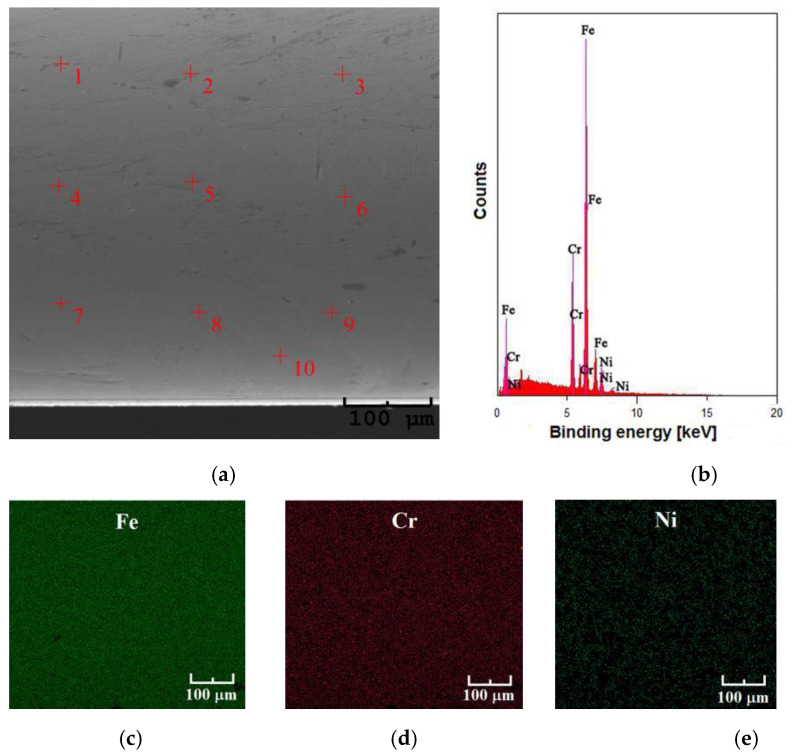
Chemical composition of the commercial Fe–Cr–Ni steel: (**a**) SEM image of the surface morphology in the selected microregion subjected to EDS analysis with measuring points 1–10; (**b**) EDS spectrum in the examined microregion; (**c**) surface distribution map of Fe; (**d**) surface distribution map of Cr; (**e**) surface distribution map of Ni.

The control analysis of the local chemical composition on the surface of the studied steel was carried out by the EDS method in selected microregions at 10 measuring points (Figure 3a). The exemplary EDS spectrum is shown in Figure 3b. Qualitative EDS analysis carried out based on the binding energy of characteristic peaks allowed to identification the presence of elements of an atomic number equal to 24, 26, and 28, i.e., chromium, iron, and nickel, respectively. Quantitative EDS analysis for the Fe–Cr–Ni steel showed the content of Fe 74.4(7) at.%, Cr 18.4(4) at.%, and Ni 7.2(4) at.%. The obtained results remain in very good compliance with the chemical composition given by the steel manufacturer.

Maps of the surface distribution of the identified elements are presented in Figure 3c–e. The obtained maps indicate that in the observed microregions Fe, Cr, and Ni are evenly distributed on the surface of the steel. Fe appears in the largest amount and Ni in the smallest one. There is also a lack of microcracks, scratches, or other damage on the surface of the Fe–Cr–Ni steel, in which corrosion cells could be initiated.

### 3.2. Micromechanical Properties of the Fe–Cr–Ni Steel

The Vickers microhardness measurements were carried out on two samples of the Fe–Cr–Ni steel 25 mm long at six measuring points, and the obtained results are shown in Table 2.

The average Vickers microhardness value for the Dentaurum Remanium^®^ archwire is 540.6 μHV_0.3_. Analysis of the Vickers microhardness of selected orthodontic archwires in the literature showed comparable values for stainless steel like GAC^®^ (578.56 μHV_0.2_), Ortho Organizers^®^ (555.67 μHV_0.2_), Ormco^®^ (609.78 μHV_0.2_), Dentaurum^®^ (579.33 μHV_0.2_), and 3M Unitek^®^ (555.33 μHV_0.2_) [31]. A much smaller value of 340.22 μHV_0.2_ was determined in the case of β-titanium alloy (TMA, Ormco^®^), similarly as for Ni–Ti alloys like Neo Sentalloy^®^ GAC (316.33 μHV_0.2_), Nitanium Ortho Organizers^®^ (403.22 μHV_0.2_), Ni–Ti Ormco^®^ (419.78 μHV_0.2_), Remetitan Dentaurum^®^ (400.22 μHV_0.2_), and Nitinol Classic 3M Unitek^®^ (444.22 μHV_0.2_). The obtained results show that the tested Fe–Cr–Ni steel is characterized by higher resistance to scratching and abrasive wear in comparison with β−titanium and Ni–Ti alloys. The strong correlation between microhardness and strength can be also expected. The micromechanical properties of the tested steel confirm the usefulness of the Dentaurum Remanium^®^ archwire in orthodontic treatment.

### 3.3. In Vitro Tests of Open-Circuit Potential

**Figure 4:**
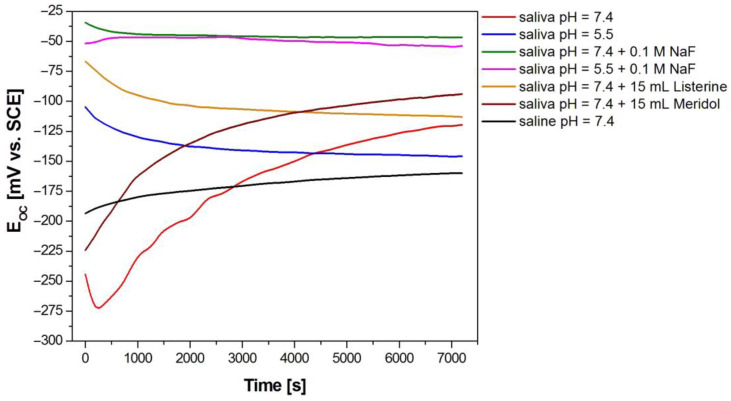
Open-circuit potential for the Fe–Cr–Ni electrode in the biological environment at 37 °C.

In vitro tests of the corrosion resistance of the Dentaurum Remanium^®^ archwire began with open-circuit potential measurements in the biological environment. The obtained curves E_OC_ = f(t) were used to pre-assess the impact of the modification of the artificial saliva solution on the corrosion resistance of the tested archwire. Figure 4 shows the results of measuring the E_OC_ for the Fe–Cr–Ni electrode in the solution of artificial saliva with physiological and acidic pH before and after modification with Listerine^®^ and Meridol^®^ mouthwashes and sodium fluoride. Comparatively, the curve showing the E_OC_ dependence on the time of immersion in the saline solution is also included.

After 7200 s from the immersion of the Fe–Cr–Ni electrode in the biological environment, the ion–electron equilibrium at the interface of electrode|electrolyte is observed. The obtained results indicate that the modification of the artificial saliva solution with mouthwashes and NaF increases the E_OC_ value compared to the solution of artificial saliva with both physiological and acidic pH. Moreover, the effect of fluoride ions originating from NaF on an increase in E_OC_ is higher compared to both types of mouthwashes used. The highest value E_OC_ equal to –47(5) mV is determined in the solution of artificial saliva with pH = 7.4 after adding sodium fluoride. The open-circuit potential value is the smallest for the Fe–Cr–Ni electrode in the saline solution, E_OC_ = –160(7) mV. The stable E_OC_ values were considered in further electrochemical measurements as approximate corrosion potential (E_cor_) values.

### 3.4. Mechanism and Kinetics of Pitting Corrosion

Alternating current (AC) measurements by the EIS method were performed to determine the effect of modifying the artificial saliva solution on the mechanism and kinetics of electrochemical corrosion of the Remanium^®^-type orthodontic archwire. To explain the impedance behavior of the examined Fe–Cr–Ni steel, the concept of equivalent electrical circuits was used. In the approximation procedure, the capacitor has been replaced by a constant phase element (CPE) representing a “leaky” capacitor, which has a non-zero real and imaginary component. The CPE impedance (${\hat{Z}}_{CPE}$) is defined as [32]:

(1) $${\hat{Z}}_{CPE}=\frac{1}{T{\left(j\omega \right)}^{\phi }}.$$

In Equation (1), T expressed in F cm^−2^ s^ϕ−1^ is a capacitive parameter, which depends on the potential of the electrode, and ϕ corresponds to the angle of rotation of the purely capacitive line on the plots of the complex plane, α = 90°(1 − ϕ).

**Figure 5:**
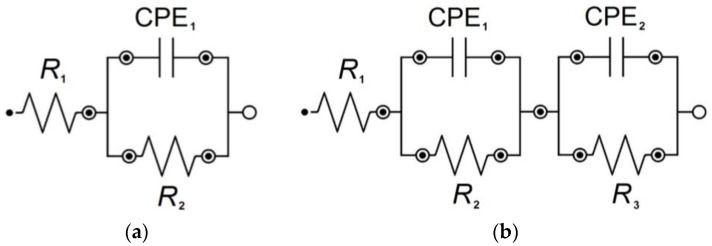
Equivalent electrical circuit for the pitting corrosion of Fe–Cr–Ni steel in the biological environment: (**a**) CPE1 model; (**b**) CPE2 model.

For the interpretation of experimental EIS spectra, for which a one-time constant was observed in the electrical circuit, the CPE1 model presented in Figure 5a was used. The CPE1 model is consisting of a resistor (R_1_) connected in series with one parallel CPE_1_-R_2_ system. This model produces one semicircle in the Nyquist diagram in the entire range of frequencies tested [2,3,7,12,32]. In the CPE1 model, the R_1_ element reflects the resistance of the electrolyte constituting the corrosion environment. CPE_1_ determines the capacity of the electrical double layer for the interface of metal covered with an oxide layer and corrosion environment. R_2_ resistor reproduces the resistance of the charge transfer resistance (R_ct_) through this interface.

CPE2 model for the pitting corrosion of Fe–Cr–Ni steel in the biological environment shown in Figure 5b was used to interpret the experimental EIS spectra with two-time constants in the electrical circuit. The CPE2 model is consisting of a resistor (R_1_) connected in series with two consecutive parallel CPE_1_-R_2_ and CPE_2_-R_3_ systems. In the case of the CPE2 model, two semicircles are observed as a frequency function in the Nyquist diagram [2,7,32]. In this model, the R_1_ element is due to the electrolyte resistance. The CPE_1_-R_2_ system is considered for an inner oxide layer with barrier properties. CPE_1_ and R_2_ correspond to the capacitance and resistance of the barrier oxide layer adjacent to the metallic substrate, respectively. The CPE_2_-R_3_ system is associated with an outer oxide layer with the presence of pits on the surface. CPE_2_ denotes the pit wall capacitance. R_3_ is the additional resistance of the electrolyte inside the pit.

**Figure 6:**
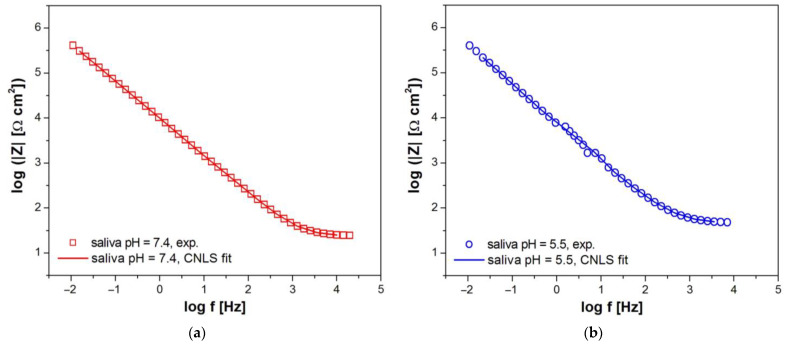

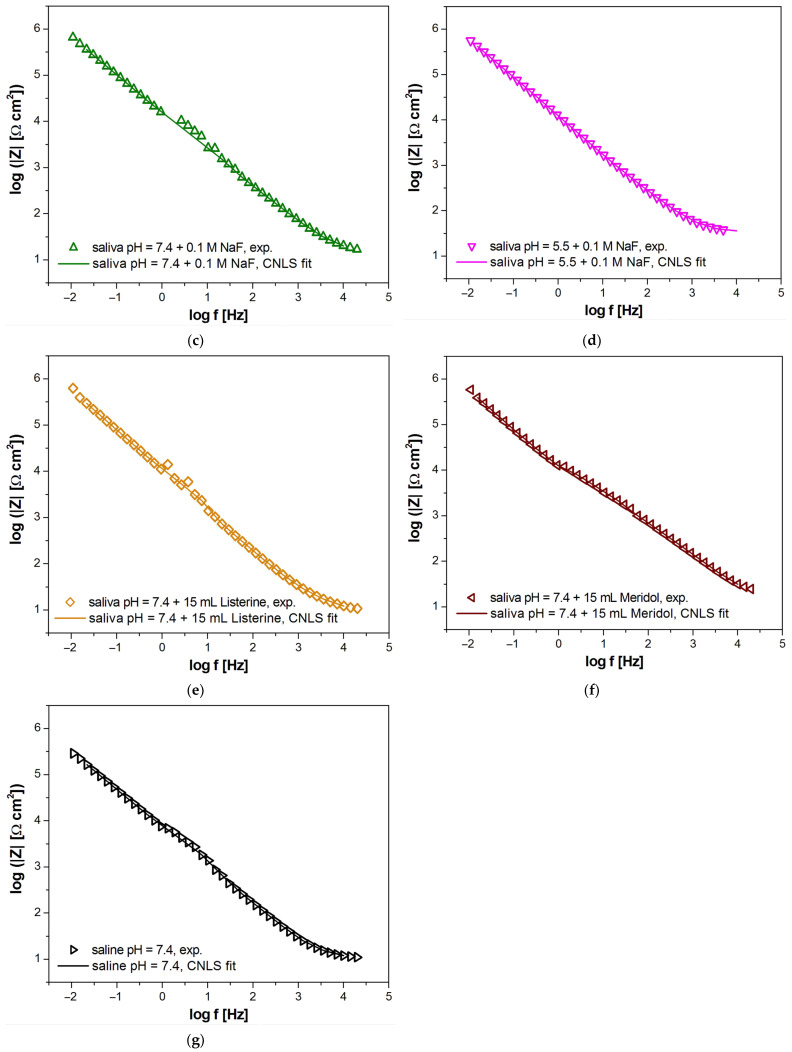
Experimental (symbols) and fitted (continuous lines) Bode diagrams in the form of log|Z| = f(log f) for the pitting corrosion of Fe–Cr–Ni steel at 37 °C in: (**a**) saliva pH = 7.4; (**b**) saliva pH = 5.5; (**c**) saliva pH = 7.4 + 0.1 M NaF; (**d**) saliva pH = 5.5 + 0.1 M NaF; (**e**) saliva pH = 7.4 + 15 mL Listerine^®^; (**f**) saliva pH = 7.4 + 15 mL Meridol^®^; (**g**) saline pH = 7.4.

**Figure 7:**
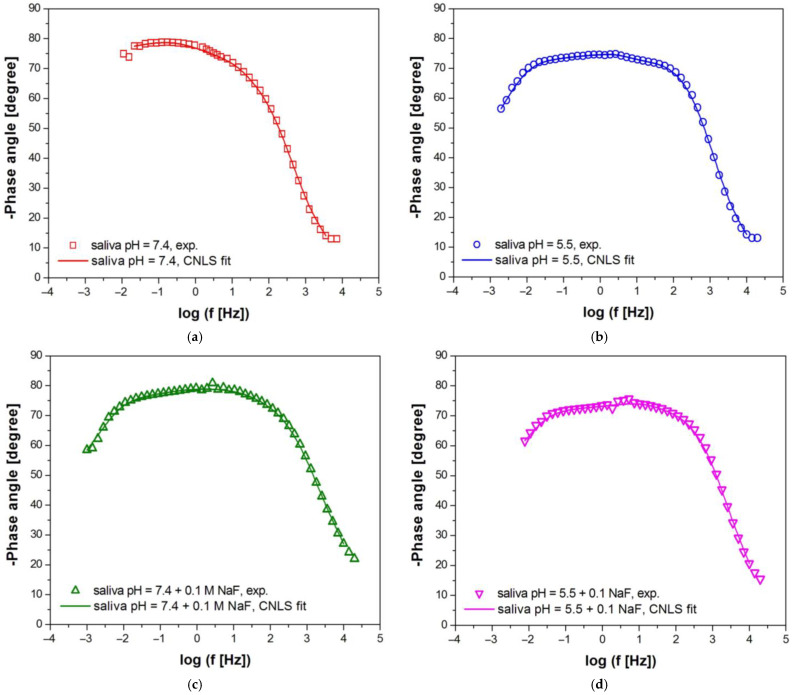

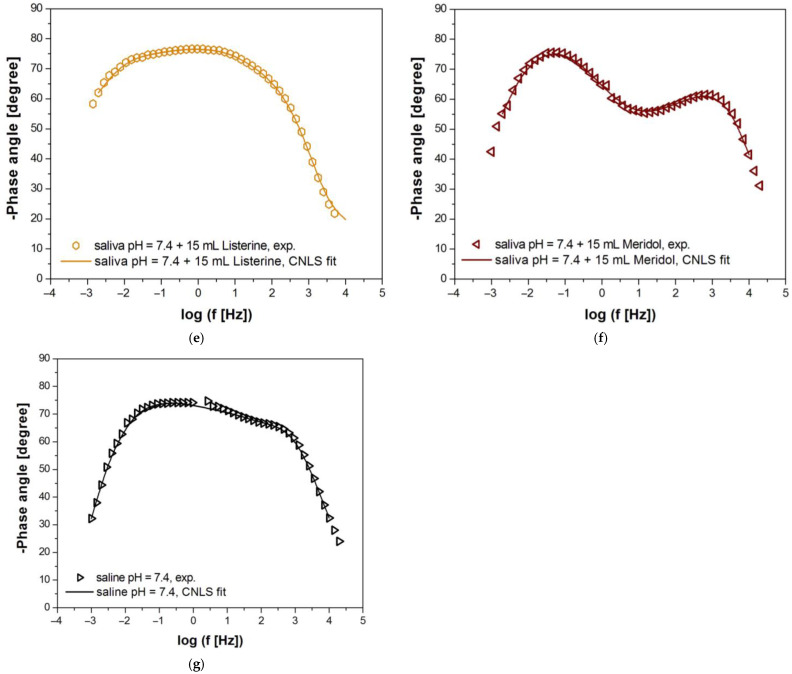
Experimental (symbols) and fitted (continuous lines) Bode diagrams in the form of phase angle versus frequency logarithm for the pitting corrosion of Fe–Cr–Ni steel at 37 °C in: (**a**) saliva pH = 7.4; (**b**) saliva pH = 5.5; (**c**) saliva pH = 7.4 + 0.1 M NaF; (**d**) saliva pH = 5.5 + 0.1 M NaF; (**e**) saliva pH = 7.4 + 15 mL Listerine^®^; (**f**) saliva pH = 7.4 + 15 mL Meridol^®^; (**g**) saline pH = 7.4.

Figure 6 and Figure 7 show experimental (symbols) and fitted (continuous lines) Bode diagrams for the pitting corrosion process of Fe–Cr–Ni steel in the biological environment at 37 °C. For the approximation of EIS spectra obtained in the solution of artificial saliva before and after modification with NaF and Listerine^®^ mouthwash, the CPE1 model was used (see Figure 5a). Only a one-time constant was observed in those electrical circuits. The CNLS fitting of EIS spectra recorded in the solution of artificial saliva with the addition of Meridol^®^ mouthwash and saline of physiological pH was carried out using the CPE2 model (see Figure 5b). Two-time constants were observed in those electrical circuits. Very good quality of the CNLS fitting for EIS experimental data was observed. In all Bode diagrams in the form of log|Z| = f (log f), a slope of about −1 is observed in the medium frequency range (Figure 6). There are also slight differences in log|Z| value at the lowest measuring frequencies, which indicates a change in the corrosion resistance of the Fe–Cr–Ni electrode to pitting as a result of the modification of the artificial saliva solution. It can be observed that the change trend of log|Z|at f = 10 mHz is the same as in the case of the E_OC_ (see Figure 4). The highest log|Z|_f=10 mHz_ equal to 5.82(64) Ω cm^2^ is obtained in the solution of artificial saliva with pH = 7.4 after modification using NaF. The log|Z|_f=10 mHz_ of 5.46(60) Ω cm^2^ takes the smallest value in the saline solution. Such a behavior of AC impedance for the Fe–Cr–Ni electrode could be caused by an increased content of aggressive Cl^–^ ions, accelerating pitting corrosion.

Bode diagrams presented in Figure 7 show that the highest maximum values of the phase angle close to −80° are observed for the Fe–Cr–Ni electrode in the solution of artificial saliva with pH = 7.4 with the addition of NaF. A slight decrease in the value of the phase angle is visible in the solution of artificial saliva with pH = 7.4 before and after addition of Listerine^®^ and Meridol^®^ mouthwashes, with pH = 5.5 before and after modification using NaF, and saline with pH = 7.4. The decrease in the phase angle value indicates an increase in the conductivity of the Fe–Cr–Ni electrode. High values of |Z| _f→0_ (Figure 6) and phase angle (Figure 7) testify to the capacitive behavior of the Fe–Cr–Ni electrode, which shows high resistance to pitting in all tested corrosive solutions.

Table 3 presents the average values of four parameters (R_1_, CPE_1_-T, CPE_1_-ϕ, R_2_) describing the equivalent electrical circuit shown in Figure 5a, which was used for CNLS fitting of experimental EIS data obtained at the E_cor_ for the Fe–Cr–Ni electrode in the biological environment at 37 °C. Table 4 shows the average values of seven parameters (R_1_, CPE_1_-T, CPE_1_-ϕ, R_2_, CPE_2_-T, CPE_2_-ϕ, R_3_) determined for the equivalent electrical circuit presented in Figure 5b, which was used in the CNLS fitting procedure of EIS spectra recorded at the E_cor_ for the Fe–Cr–Ni electrode in the artificial saliva of pH = 7.4 with the addition of 15 mL Meridol^®^ and saline of pH = 7.4 at 37 °C.

The R_1_ parameter depends on the chemical composition of the solution and reaches a value in the range from 4.79(24) Ω cm^2^ in the saline solution with pH = 7.4 (Table 3) to 20.79(41) Ω cm^2^ in the solution of artificial saliva with pH = 5.5 modified using sodium fluoride (Table 4). The capacitive behavior of the tested electrochemical systems indicates that all values of the CPE_1_-T parameter are of the order of 10^−5^ F cm^−2^ s^ϕ−1^. These are typical capacitance parameter values for metallic biomaterials in the biological environment [2,3,7,32]. The smallest value of the parameter CPE_1_-T of 1.71(39) × 10^−5^ F cm^−2^ s^ϕ−1^ was determined in the solution of artificial saliva with pH = 7.4 after modification using NaF (Table 3), which indicates the smallest surface of the Fe–Cr–Ni electrode, devoid of extensive and deep pits. The CPE_1_-ϕ values reveal a significant deviation from a possible maximum value equal to 1 (Table 3 and Table 4). This empirical parameter is associated with the presence of heterogeneities of physical, chemical, and geometrical nature [32]. All values of the R_2_ are of the order of 10^6^ Ω cm^2^ (Table 3 and Table 4). The highest value of the R_2_ equal to 7.47(18) × 10^6^ Ω cm^2^ was obtained in the solution of artificial saliva with pH = 7.4 enriched with NaF (Table 3). The lowest value of this kinetic parameter equal to 1.28(90) × 10^6^ Ω cm^2^ was determined in the saline solution (Table 4). The physicochemical meaning of the R_2_ is due to the ongoing pitting corrosion. It can be expected that the smaller the value of R_2_, the faster the kinetics of the pitting corrosion. The obtained R_2_ values indicate that the self-passive oxide layer on the surface of the Fe–Cr–Ni electrode shows the strongest barrier properties for aggressive chloride ions in the solution of artificial saliva at physiological pH containing F^−^ ions. The R_3_ determined in the solution of saliva with pH = 7.4 modified with Meridol^®^ mouthwash and saline has a value of three orders lower compared to the R_2_ (Table 4). The corresponding CPE_2_-T values are increased in relation to the CPE_1_-T, and the CPE_2_-ϕ are characteristic of the lower values in comparison with the CPE_1_-ϕ (Table 4). Such behavior points that the exposed surface area of the Fe–Cr–Ni electrode increases as a result of uncovering pits. These results confirm that the self-passive layer is more conductive and its resistance to pitting corrosion is weakened.

### 3.5. Susceptibility to Pitting Corrosion

**Figure 8:**
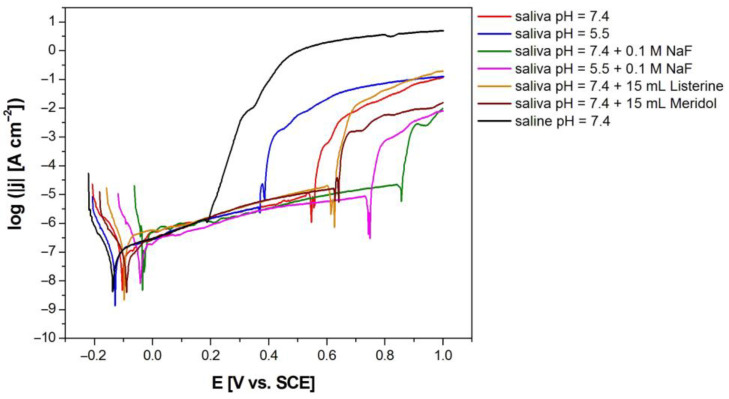
Anodic polarization curves of the Fe–Cr–Ni electrode obtained at the polarization rate of 1 mV s^−1^ in the biological environment at 37 °C.

The effect of modification of the artificial saliva solution on the susceptibility of the Dentaurum Remanium^®^ archwire to pitting corrosion was determined based on the anodic polarization curves recorded by the potentiodynamic method (Figure 8). Potentiodynamic characteristics revealed the passive behavior of the Fe–Cr–Ni electrode in the biological environment at 37 °C. It can be observed that the modification of artificial saliva solutions of physiological and acidic pH causes the anodic polarization curves to shift in the direction of anodic potentials, which indicates an increase in the corrosion resistance of the Fe–Cr–Ni electrode. The most cathodic values of potentials testifying to the lowest corrosion resistance of the tested electrode are observed in the case of the potentiodynamic curve, which was registered in the saline solution of pH = 7.4.

All obtained curves log|j| = f(E) have the same shape and are characterized by the lack of a clear area where active dissolution of the self-passive oxide layer proceeds. The E_cor_ and the corresponding j_cor_ are in the passive area of the anodic polarization curves. The E_cor_ shows the same nature of changes and similar values as E_OC_ (Table 5). The j_cor_ reveals values in the range from 1.35(27) to 8.13(92) × 10^−9^ A cm^−2^ (Table 5).

At potentials lower than the E_cor_, the Fe–Cr–Ni electrode shows resistance to pitting corrosion. At potentials above the E_cor_, the process of anodic dissolving of the self-passive oxide layer takes place. The obtained densities of passive currents are typical of metallic biomaterials in the biological environment [2,3,7,32]. After exceeding the passive range, a rapid increase in the density of the anodic current is observed as a result of breaking the oxide layer. Breakdown potential (E_bd_) and corresponding values of breaking current density (j_bd_) are presented in Table 5. The analysis of the results obtained showed that the E_bd_ takes values from 0.178(4) V in the saline solution with pH = 7.4 to 0.843(17) V in the solution of artificial saliva with pH = 7.4 modified using sodium chloride. Thus, sodium fluoride, which is a component of toothpaste, reduces the susceptibility of the Dentaurum Remanium^®^ archwire to pitting corrosion. The fastest breakdown of the oxide layer will occur in the solution of sodium chloride with the highest content of aggressive Cl^−^ ions.

**Figure 9:**
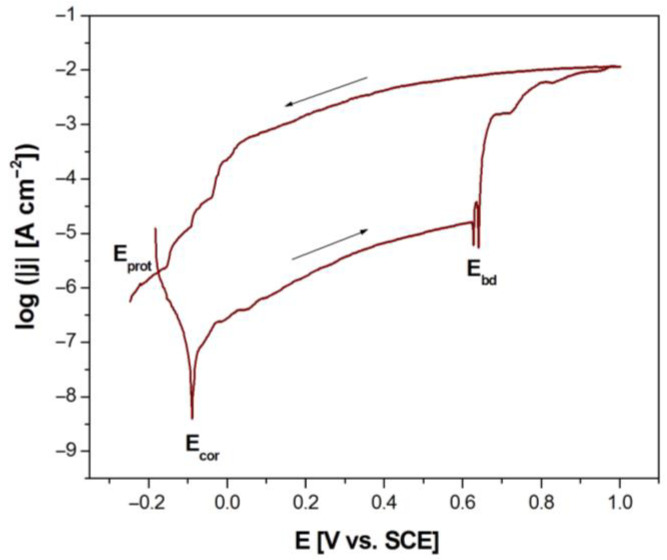
Cyclic polarization curves of the Fe–Cr–Ni electrode obtained at the polarization rate of 1 mV s^−1^ in the artificial saliva at pH = 7.4 with the addition of 15 mL Meridol^®^ at 37 °C.

The protective potential (E_prot_) and corresponding protective current density (j_prot_) were determined using cyclic polarization curves. Figure 9 shows an exemplary cyclic polarization curves of the Fe–Cr–Ni electrode obtained at the polarization rate of 1 mV s^−1^ in the artificial saliva at pH = 7.4 with the addition of 15 mL Meridol^®^ at 37 °C. The difference between E_prot_ and E_bd_ shows a strong susceptibility of the tested archwire to pitting corrosion in each corrosion environment used (Table 5).

### 3.6. Assessment of Corrosive Damage

**Figure 10:**
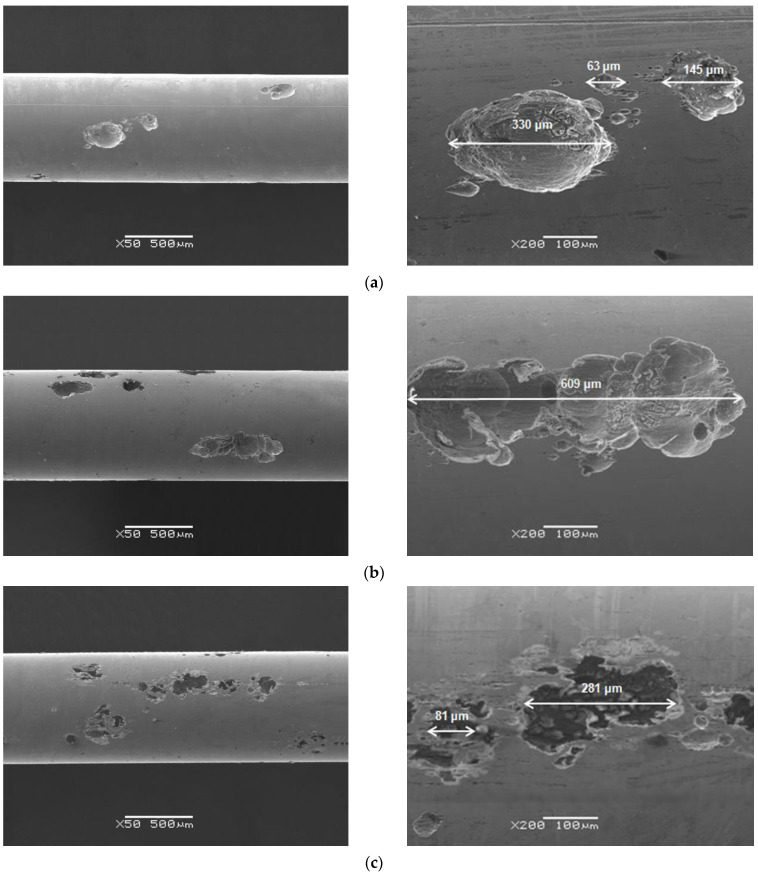
SEM image of the Dentaurum Remanium^®^ archwire surface after potentiodynamic tests in: (**a**) saliva pH = 7.4; (**b**) saliva pH = 5.5; (**c**) saline pH = 7.4.

**Figure 11:**
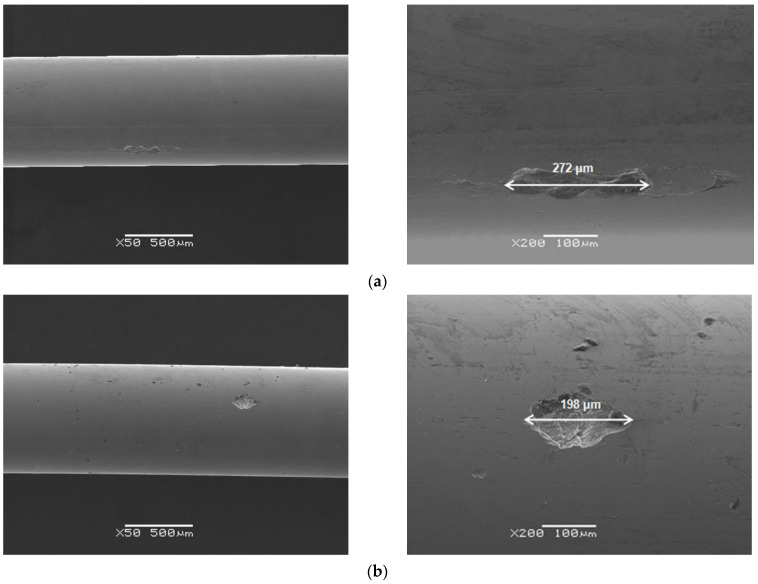
SEM image of the Dentaurum Remanium^®^ archwire surface after potentiodynamic tests in: (**a**) saliva pH = 7.4 + 0.1 M NaF; (**b**) saliva pH = 5.5 + 0.1 M NaF.

**Figure 12:**
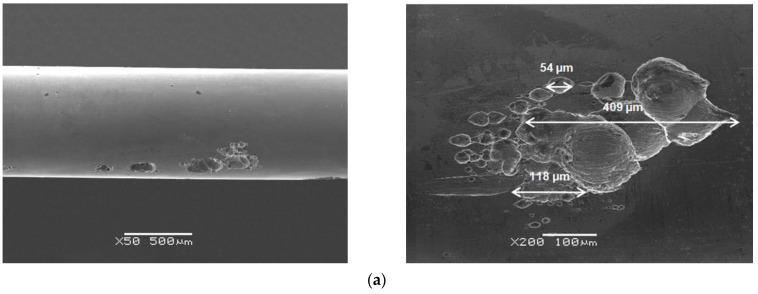

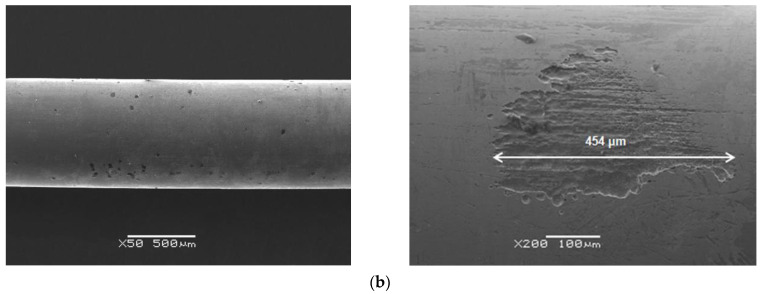
SEM image of the Dentaurum Remanium^®^ archwire surface after potentiodynamic tests in: (**a**) saliva pH = 7.4 + 15 mL Listerine^®^; (**b**) saliva pH = 7.4 + 15 mL Meridol^®^.

Microscopic observations of the surface morphology of the Dentaurum Remanium^®^ archwire after potentiodynamic tests in the biological environment were taken by SEM (Figure 10, Figure 11 and Figure 12). SEM images obtained for the Fe–Cr–Ni archwire revealed the presence of spherical pits, which were formed in the artificial saliva solutions before and after modification, and in the saline. The susceptibility of the examined archwire to pitting corrosion depends on the chemical composition of the solution. A greater susceptibility to the corrosion of the archwire was observed in the solution of artificial saliva with acidic pH than physiological (Figure 10). It is also seen that after adding sodium fluoride (Figure 11) and mouthwashes (Figure 12) to the artificial saliva solution (Figure 10a,b), the number and the size of diagnosed pits on the archwire surface decreased significantly. The slightest susceptibility to the formation of pits was demonstrated by the archwire after corrosion tests in the solution of artificial saliva with pH = 7.4 enriched with sodium fluoride (Figure 11a). A single, small, and shallow pit is visible in the examined microregion on the archwire surface. The most intensive process of pitting corrosion of the archwire took place in the solution of physiological saline, on the surface of which numerous deep pits were observed (Figure 10c).

The results of the quantitative analysis of the chemical composition of the Dentaurum Remanium^®^ archwire after corrosion resistance research obtained by the EDS method are presented in Table 6, Table 7, Table 8, Table 9, Table 10, Table 11 and Table 12.

EDS analysis showed that the greatest impoverishment of the Fe–Cr–Ni steel in Fe, Cr, and Ni elements occurred in the saline solution as a result of the aggressive interaction of the Cl^−^ ions on the self-passive oxide layer (Table 8). The slightest impoverishment in alloying elements occurs in the artificial saliva solution enriched with sodium fluoride with pH = 7.4, which indicates the protective effect of F^−^ ions (Table 9). It is observed that in micro-areas with pits created, the largest reduction in iron content occurred in relation to the remaining alloying elements (Table 6, Table 7, Table 8, Table 9, Table 10, Table 11 and Table 12). The cathodic process occurring in the deaerated biological environment at the E_cor_ may be the reduction of hydrogen ions in accordance with Equation (2):

(2) $$H^{+}+e\to \frac{1}{2}H_{2}.$$

It can be assumed that the anodic process taking place at the E_cor_ is the dissolution of the alloying component. Since the chromium oxidation process is at a high rate, it cannot be a reaction that limits the rate of the anodic process. Therefore, it can be hypothesized that the occurring process is the dissolution of iron. This process may occur according to the mechanism, in which the stage limiting the rate of the whole process is the stage based on one-electron exchange in accordance with Equation (3):

(3) $$Fe{\left(OH\right)}_{ads}\to FeOH^{+}+e.$$

Oxygen, which is associated with corrosion products formed as a result of secondary reactions, was also identified in the interior of the pits visible in Figure 10, Figure 11 and Figure 12. It is observed that the greater the impoverishment in the alloying elements, the greater the oxygen content in the interior of the pit (Table 6, Table 7, Table 8, Table 9, Table 10, Table 11 and Table 12).

**Figure 13:**
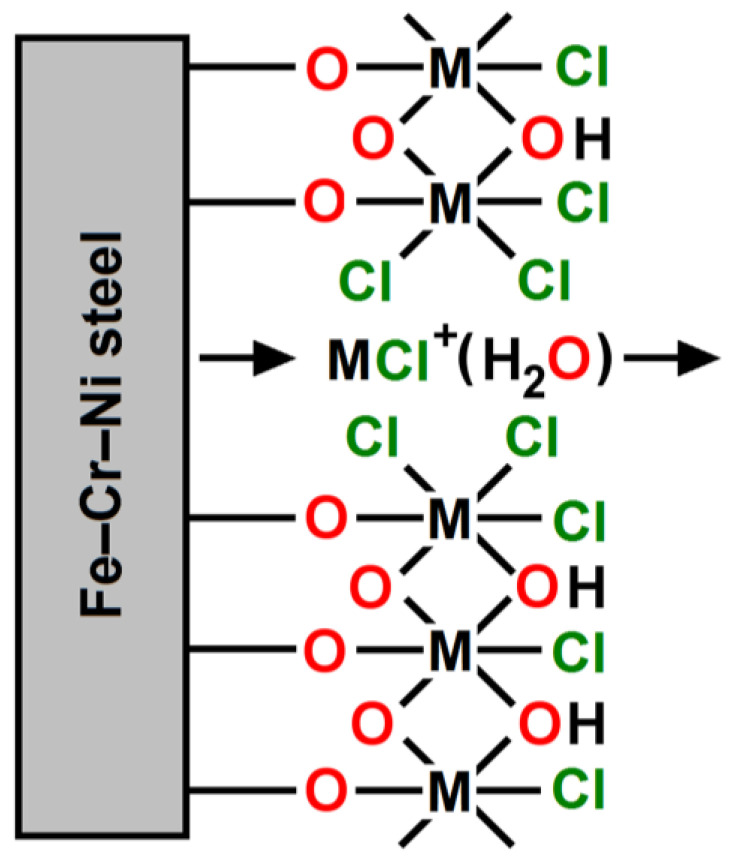
Model for pitting corrosion of the self-passive oxide layer on the Dentaurum Remanium^®^ archwire surface in a biological environment containing chloride ions.

The mechanism of initiation and development of pits in the self-passive oxide layer on the surface of the Dentaurum Remanium^®^ archwire in the biological environment containing chloride ions is schematically presented in Figure 13.

Pitting corrosion of the Dentaurum Remanium^®^ archwire in the biological environment is a local type of corrosion. The emerging pits are invisible at the stage of formation. The weight loss of the archwire is small, but progressive local damage can lead to destruction as a result of the perforation of the element. In the process of pitting corrosion of the archwire, two stages one can distinguish, namely the initiation of the pit and the growth of the existing pit. The initiation of the pit occurs at the localized areas where the self-passive oxide layer is the weakest, i.e., at micro-areas with mechanical damage and at the border of grains. Pit embroidery is preceded by the adsorption of aggressive chloride ions on the surface of the Fe–Cr–Ni steel, which penetrate through the self-oxide layer. The embryonic time depends on the quality of the material and the corrosion environment. The pitting corrosion model of the passive stainless steel in an acidic solution containing chloride ions was proposed by Okamoto [33]. The most important parameter responsible for the high resistance of the tested 18-8 stainless steel to pitting corrosion is the amorphous nature of the passive oxide layer with bound water included. In the Okamoto model, water molecules are replaced by chloride ions and there is a combination of chloride ions with metal, which makes it difficult to build the metal ions into the passive layer, and facilitates their transition to the solution. The passive layer is not repassivated. The development of the existing pit occurs through the growth of the pits arising during the incubation period and the creation of new pits. The shape and size of the pits vary depending on the conditions of the pitting corrosion, the type of material, and the polarization conditions. The surface of the pit is an anode, while in the interior of the pit, the metal is dissolved. The entertainment of the pit is a cathode, so there is an oxygen reduction or hydrogen evolution. In the interior of the pit, the concentration of aggressive ions increases and the pH in the acidic direction is reduced. A layer of corrosion products is formed at the bottom of the pit. The replacement of the electrolyte between the pit and the corrosion environment occurs through the holes and pores of the passive layer. The composition, thickness, and porosity of the passive layer affect the size and rate of the development of pits. Fluoride anions facilitate the formation of the chrome oxide layer, which means that the corrosion resistance of Cr-containing alloys in an environment containing fluoride ions is higher [34]. Contents of 18%at. Cr in stainless steel with self-passivation ability ensures that the Remanium^®^-type orthodontic archwire demonstrates corrosion resistance in the biological environment comparable to the resistance of precious metal alloys [22].

### 3.7. Mechanical Properties of the Fe–Cr–Ni Steel

**Figure 14:**
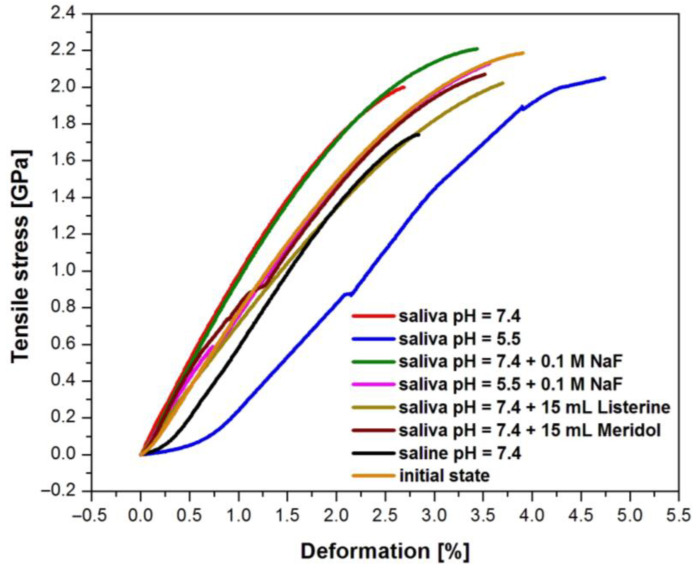
Dependence of tensile stress as a deformation function for the Dentaurum Remanium^®^ archwire before and after corrosion investigation in the tensile test.

Figure 14 shows experimental curves illustrating the dependence of tensile stress as a deformation function for the samples of the Dentaurum Remanium^®^ orthodontic archwire obtained in the tensile test before and after corrosion investigation. Good repeatability of the results was observed.

Table 13 includes the values of parameters determined in the tensile test of the Dentaurum Remanium^®^ archwire, such as tensile strength (R_m_) and deformation for R_m_.

Strength measurements in the tensile test showed that the analyzed Fe–Cr–Ni steel is characterized by high tensile strength. This is evidenced by the Young’s modulus equal to 134.24 GPa. This value is similar to Young’s modulus for titanium and titanium alloys (E ≈ 105 GPa) [35]. The average deformation for R_m_ is 2.70% for the archwire at the initial state. These values indicate that the Fe–Cr–Ni steel is a plastic material, undergoing deformations and is ideally suited for use on orthodontic arches. The average deformation for R_m_ determined for the same dental material after corrosion tests revealed the highest values up to 4.00%, depending on the corrosive agent used. The obtained results indicate a weakening of the mechanical properties of the tested archwire due to corrosion damage.

## 4. Conclusions

The Remanium^®^-type orthodontic archwire containing in its chemical composition 74.4(7) at.%, Cr 18.4(4) at.%, and Ni 7.2(4) at.% is a bi-phase alloy showing a mixed austenite/ferrite structure. The analyzed Fe–Cr–Ni steel is characterized by high tensile strength and the average Vickers microhardness, indicating that the Fe–Cr–Ni steel is a plastic material, undergoing deformations and suited for use on orthodontic archwires.

The modification of the artificial saliva solution with sodium fluoride and mouthwashes causes an increase in the corrosion resistance of the tested archwire in comparison with the artificial saliva solution of both physiological and acidic pH. The highest corrosion resistance of the self-passive oxide layer on the surface of the Fe–Cr–Ni electrode was revealed in the solution of artificial saliva at physiological pH containing F^−^ ions, while the lowest kinetics of pitting corrosion was determined in the saline solution due to the presence of aggressive chloride ions. The quantitative assessment of the resistance to pitting corrosion based on the EIS measurements allowed to determine the impedance of the interface Fe–Cr–Ni steel | electrolyte, which, depending on the corrosion environment, can be described with the CPE1 or CPE2 equivalent electrical circuit model. The potentiodynamic characteristics of the Fe–Cr–Ni steel showed the passive behavior and the decrease in susceptibility to pitting corrosion in the presence of fluoride anions facilitating the formation of a passive layer and mouthwashes added in the artificial saliva solution. The mechanism of initiation and growth of pits in the self-passive oxide layer on the surface of the Fe–Cr–Ni steel in the biological environment containing chloride ions was interpreted based on the Okamoto model. It was found that the mechanical properties of the tested archwire after corrosion tests were weakened. The obtained results confirmed that the Dentaurum Remanium^®^ archwire meets the criteria of the resistance to pitting corrosion for dental materials for orthodontic wires.

## Figures and Tables

**Table 1:** Chemical composition of the artificial saliva [25].

Component	Concentration [g dm^−3^]
Na_2_HPO_4_	0.260
KH_2_PO_4_	0.200
NaHCO_3_	1.500
KSCN	0.330
NaCl	6.700
KCl	1.200

**Table 2:** Results of the Vickers microhardness for the Dentaurum Remanium^®^ archwire.

Measurement Number	Sample 1 [μHV_0.3_]	Sample 2 [μHV_0.3_]
1	413.0	467.0
2	555.4	593.5
3	542.8	579.5
4	534.7	574.3
5	596.7	604.4
6	518.8	506.9
Average value	540.6	
Standard deviation	19.4	

**Table 3:** The average values of parameters obtained as a result of the CNLS fitting of the experimental EIS spectra for the Fe–Cr–Ni electrode and the CPE1 model for the pitting corrosion (see Figure 5a) in the biological environment at 37 °C.

Electrolyte Type	R_1_ (Ω cm^2^)	CPE_1_-T (F cm^−2^ s^ϕ−1^)	CPE_1_-ϕ	R_2_ (Ω cm^2^)
Saliva pH = 7.4	7.87(64)	3.03(12) × 10^−5^	0.855(7)	2.78(68) × 10^6^
Saliva pH = 5.5	10.14(28)	8.23(14) × 10^−5^	0.826(4)	1.42(77) × 10^6^
Saliva pH = 7.4 + 0.1 M NaF	7.94(63)	1.71(39) × 10^−5^	0.813(4)	7.47(18) × 10^6^
Saliva pH = 5.5 + 0.1 M NaF	20.79(41)	2.78(23) × 10^−5^	0.836(2)	6.12(69) × 10^6^
Saliva pH = 7.4 + 15 mL Listerine	15.36(23)	4.24(39) × 10^−5^	0.855(2)	2.97(88) × 10^6^

**Table 4:** The average values of parameters as a result of the CNLS fitting of the experimental EIS spectra for the Fe–Cr–Ni electrode and the CPE2 model for the pitting corrosion (see Figure 5b) in the biological environment at 37 °C.

Electrode Type	R_1_ (Ω cm^2^)	CPE_1_-T (F cm^−2^ s^ϕ−1^)	CPE_1_-ϕ	R_2_ (Ω cm^2^)	CPE_2_-T (F cm^−2^ s^ϕ−1^)	CPE_2_-ϕ	R_3_ (Ω cm^2^)
Saliva pH = 7.4 + 15 mL Meridol	4.81(36)	4.16(70) × 10^−5^	0.895(11)	3.01(44) × 10^6^	1.02(59) × 10^−4^	0.602(6)	2.47(48) × 10^3^
Saline pH = 7.4	4.79(24)	4.50(19) × 10^−5^	0.867(15)	1.28(90) × 10^6^	9.33(15) × 10^−5^	0.787(7)	2.78(11) × 10^3^

**Table 5:** The key potential-current parameters for the Fe–Cr–Ni electrode determined based on the anodic polarization curves in the biological environment at 37 °C (see Figure 8).

Electrolyte Type	E_cor_ (mV)	j_cor_ (A cm^−2^)	E_bd_ (mV)	j_bd_ (A cm^−2^)	E_prot_ (mV)	j_prot_ (A cm^−2^)
Saliva pH = 7.4	–104(21)	4.68(94) × 10^−9^	534(11)	1.15(23) × 10^−5^	–205(22)	1.05(22) × 10^−5^
Saliva pH = 5.5	–128(26)	1.35(27) × 10^−9^	370(7)	2.29(46) × 10^−6^	–36(9)	2.09(24) × 10^−7^
Saliva pH = 7.4 + 0.1 M NaF	–34(7)	4.66(83) × 10^−9^	843(17)	2.19(44) × 10^−5^	121(12)	2.72(25) × 10^−6^
Saliva pH = 5.5 + 0.1 M NaF	–42(8)	8.13(92) × 10^−9^	732(15)	8.71(74) × 10^−6^	–99(13)	1.60(19) × 10^−6^
Saliva pH = 7.4 + 15 mL Listerine	–97(19)	2.14(43) × 10^−9^	602(12)	2.04(41) × 10^−5^	–155(18)	6.92(32) × 10^−6^
Saliva pH = 7.4 + 15 mL Meridol	–89(18)	3.98(80) × 10^−9^	624(13)	1.62(32) × 10^−5^	–162(19)	2.25(20) × 10^−6^
Saline pH = 7.4	–137(27)	4.17(83) × 10^−9^	178(4)	1.38(28) × 10^−6^	–210(19)	1.74(18) × 10^−6^

**Table 6:** Chemical composition of the Dentaurum Remanium^®^ archwire after corrosion tests in saliva pH = 7.4 at 37 °C.

Element	Saliva pH = 7.4		Standard Deviation	
	Sample	Pit	Sample	Pit
Fe	73.7	64.5	0.8	5.6
Cr	19.0	17.3	1.0	0.9
Ni	7.3	6.7	0.2	0.6
O	-	11.6	-	7.1

**Table 7:** Chemical composition of the Dentaurum Remanium^®^ archwire after corrosion tests in saliva pH = 5.5 at 37 °C.

Element	Saliva pH = 5.5		Standard Deviation	
	Sample	Pit	Sample	Pit
Fe	73.8	65.0	0.1	10.5
Cr	18.7	17.6	0.1	7.0
Ni	7.5	6.3	0.1	2.5
O	-	11.1	-	6.9

**Table 8:** Chemical composition of the Dentaurum Remanium^®^ archwire after corrosion tests in saline pH = 7.4 at 37 °C.

Element	Saline pH = 7.4		Standard Deviation	
	Sample	Pit	Sample	Pit
Fe	74.2	64.3	0.2	4.7
Cr	18.5	16.9	0.2	0.8
Ni	7.3	6.1	0.1	0.2
O	-	12.8	-	5.6

**Table 9:** Chemical composition of the Dentaurum Remanium^®^ archwire after corrosion tests in saliva pH = 7.4 + 0.1 M NaF at 37 °C.

Element	Saliva pH = 7.4 + 0.1 M NaF		Standard Deviation	
	Sample	Pit	Sample	Pit
Fe	74.4	68.8	0.5	5.6
Cr	18.5	17.3	0.6	0.9
Ni	7.1	6.7	0.1	0.6
O	-	7.2	-	7.1

**Table 10:** Chemical composition of the Dentaurum Remanium^®^ archwire after corrosion tests in saliva pH = 5.5 + 0.1 M NaF at 37 °C.

Element	Saliva pH = 5.5 + 0.1 M NaF		Standard Deviation	
	Sample	Pit	Sample	Pit
Fe	73.8	65.5	0.3	6.1
Cr	18.7	17.7	0.1	1.2
Ni	7.5	6.7	0.3	0.5
O	-	10.1	-	7.7

**Table 11:** Chemical composition of the Dentaurum Remanium^®^ archwire after corrosion tests in saliva pH = 7.4 + 15 mL Listerine^®^ at 37 °C.

Element	Saliva pH = 7.4 + 15 mL Listerine^®^		Standard Deviation	
	Sample	Pit	Sample	Pit
Fe	73.8	67.9	0.5	3.0
Cr	18.7	18.0	0.5	0.4
Ni	7.5	6.9	0.1	0.1
O	-	7.2	-	3.6

**Table 12:** Chemical composition of the Dentaurum Remanium^®^ archwire after corrosion tests in saliva pH = 7.4 + 15 mL Meridol^®^ at 37 °C.

Element	Saliva pH = 7.4 + 15 mL Meridol^®^		Standard Deviation	
	Sample	Pit	Sample	Pit
Fe	73.8	64.9	0.3	8.0
Cr	18.8	17.6	0.1	4.9
Ni	7.5	6.5	0.2	4.9
O	-	11	-	0.9

**Table 13:** Results obtained in the tensile test of the Dentaurum Remanium^®^ archwire before and after corrosion investigation.

Sample	R_m_ [GPa]	SD	Deformation for R_m_ [%]	SD
Saliva pH = 7.4	2.19	0.70	4.00	0.08
Saliva pH = 5.5	2.00	0.55	2.80	0.05
Saliva pH = 7.4 + 0.1M NaF	2.18	0.69	4.00	0.05
Saliva pH = 5.5 + 0.1M NaF	2.07	0.70	3.60	0.06
Saliva pH = 7.4 + 15 mL Listerine	2.00	0.68	4.60	0.08
Saliva pH = 7.4 + 15 mL Meridol	2.04	0.70	3.80	0.07
Saline pH = 7.4	2.21	0.67	3.50	0.07
Initial state	1.74	0.64	2.70	0.06

## Data Availability

The data presented in this study are available on request from the corresponding author.
